# Detection of enteric viruses in source and treated waters of the city of Hamadan, Iran

**DOI:** 10.7717/peerj.21173

**Published:** 2026-06-10

**Authors:** Nastaran Ansari, Zahra Mazaheri, Iman Owliaee, Morteza Abbaszadegan, Absar Alum, Razieh Amini, Farzad Beikpour, Farid Azizi Jalilian

**Affiliations:** 1 Department of Virology, Faculty of Medicine, Hamadan University of Medical Sciences, Hamadan, Iran; 2 School of Sustainable Engineering and the Built Environment, Arizona State University, Tempe, AZ, United States of America; 3 Water and Environmental Technology Center, Arizona State University, Tempe, AZ, United States of America; 4 Research Center for Molecular Medicine, Hamadan University of Medical Sciences, Hamadan, Iran; 5 St. Louis, MO, United States of America

**Keywords:** Enteric viruses, PCR, Viral prevalence, Drinking water, Source water, Iran

## Abstract

Enteric viruses are waterborne pathogens of public health concern. This study uses five different detection methods to investigate the prevalence of viral pathogens in source and treated waters of the city of Hamadan, Iran. The dataset contributes to the body of knowledge on viral occurrence from a region with limited data on enteric viruses in waters. During the summer and autumn, a total of 100 water samples were collected, including 29 from source, 24 from treated waters from two drinking water treatment plants (DWTPs), and 47 from the supply network of groundwater wells in the city of Hamadan, Iran. For each sample, five liters of water samples were collected, concentrated, and analyzed by molecular methods for the detection of enteric viruses. Samples were analyzed using two standard and three multiplex PCR methods for the detection of enteric viruses. Adenoviruses were detected in 24% of the samples, with a significant difference in positivity rates between the sites (*p* < 0.0001). Additionally, viral contamination in the source water was significantly higher compared to groundwater wells (*p* = 0.0001). Notably, statistical analysis revealed that viral occurrence was higher during the summer than in autumn. In summary, enteric viruses were detected across Hamadan’s drinking water system, indicating potential viral circulation in the environment and highlighting the need for further quantitative and infectivity assessments to evaluate community exposure risks. Notably, viral genome detection in source and treated waters of Hamadan County water supplies should be considered as an indicator of possible contamination, warranting additional monitoring by public health agencies.

## Introduction

The population growth and the increase in water consumption impact the quality and sustainability of water resources used for municipal water supplies. Water quality monitoring is essential for assessing drinking water safety and can help prevent waterborne disease outbreaks (Bouseettine et al., 2020). Surface waters are continuously contaminated with waterborne human pathogens through point and non-point sources. It is well-known that certain viruses and parasites are more resistant to water treatment processes than commonly used bacterial indicators such as *E. coli* (Abbaszadegan et al., 2008). This highlights the need to monitor viral pathogens in source and drinking water due to their higher survival rate in the environment than most intestinal bacteria (Fong & Lipp, 2005; Upfold, Luke & Knox, 2021). Studies have shown that viruses can remain infectious for up to 130 days in seawater, up to 120 days in freshwater, and up to 100 days in soil at temperatures between 20–30 °C (Bouseettine et al., 2020; La Rosa et al., 2012). The United States Environmental Protection Agency (USEPA) publishes the Contaminant Candidate List (CCL) every five years, which includes chemical and microbial contaminants based on their public health significance, occurrence, and susceptibility to treatment. Notably, viral pathogens, including Enteroviruses, Adenovirus, and Caliciviruses, have been included in all CCL published since 1998 (USEP Agency, 2022). Enteric viruses have been detected worldwide in surface water, groundwater, and drinking water distribution systems. A World Health Organization (WHO) study identified norovirus RNA in 18% of treated drinking water samples across five countries (Wu et al., 2011). Despite the low concentration of viruses in water, even a few viral particles can pose health risks due to their low infectious doses (10–100 virions) (La Rosa et al., 2012).

In Iran, limited studies have documented enteric virus contamination in water sources and related matrices, primarily in urban areas like Tehran, Hamadan, and Zahedan. For instance, a 2024 study detected NoV GI and GII (20% overall), RV (23%), AstV (16%), and AdV (12%) in vegetable samples from Tehran farms and markets, as well as in 25% of irrigation water samples in phase II, identifying sewage-contaminated irrigation as a key pre-harvest transmission route and noting the absence of HAV and HEV (Junter & Lebrun, 2017). Similarly, in Zahedan, a 2025 wastewater-based epidemiology study reported high year-round prevalence of RV (91.66%), NoV GII (100%), NoV GI (75%), and EV (83.33%) in untreated sewage, with diverse genotypes, emphasizing the utility of real-time RT-PCR for surveillance in resource-limited settings (Hamza et al., 2011). An original 2025 study in Hamadan further contextualizes these findings, reporting AdV detection in 55.1% of environmental water samples, with 50% in DWTP source water and 28.6% in treated water using nested PCR as the most sensitive method (44.9% positivity), but focusing solely on AdV as a representative enteric virus, underscoring persistence despite conventional treatments and the need for region-specific monitoring in western Iran (Abbaszadegan, Lechevallier & Gerba, 2003). However, data from western regions such as Hamadan remain scarce, with no comprehensive multiplex surveys on multiple enteric viruses in drinking water systems, despite increasing population pressures and drought risks. This gap underscores the importance of localized monitoring to inform public health strategies in Iran.

The main objective of this study was to employ different methods to investigate the prevalence of enteric viruses in source and treated waters of the city of Hamadan, Iran. The methods were used for the detection of several RNA viruses including Enteroviruses (EV), Hepatitis A virus (HAV), Hepatitis E virus (HEV), Norovirus (NoV) Genogroup I (GI) and GII, and Rotavirus Groups A and C, as well as a DNA virus, Adenovirus (AdV).

## Materials and Methods

### Study area and sample collection

The study was conducted in Hamadan, Iran, with a population of over 800,000. Sampling sites were strategically selected, including the inlet (source water) and outlet (treated water) of two main drinking water treatment plants (DWTP) and 47 groundwater wells. Shahid Beheshti DWTP and Ekbatan DWTP are Hamadan’s major DWTPs providing drinking water to the city. The Shahid Beheshti DWTP, with a nominal capacity of 1,000 L/s, primarily treats surface water sourced from the Ekbatan and Abshineh dams. Its treatment train includes pre-sedimentation, pre-ozonation, coagulation with alum and lime for pH adjustment, clarification using two Pulsators^®^, rapid sand filtration, and final chlorination. The Ekbatan DWTP, with a nominal capacity of up to 400 L/s, treats surface water primarily from the Ekbatan Dam. Its process involves primary disinfection with chlorine to address pathogens and taste/odor issues, addition of polyaluminum chloride as a coagulant, solids contact clarification using an Accelerator^®^, rapid sand filtration, and final chlorination. Groundwater from the 47 wells undergoes basic disinfection (primarily chlorination) before integration into the distribution network, without additional advanced treatment steps such as coagulation or filtration.

The drinking water supply network includes groundwater wells, from which water is disinfected prior to being added to the distribution network. The sampling campaign involved collecting samples four times a month from June to November 2018. Source and treated water samples were collected from the DWTPs, as well as from groundwater wells. Sampling during these periods was chosen due to reduced rainfall and increased water scarcity, which can lead to higher concentrations of contaminants. Monitoring water quality during these periods helps assess the impact of water scarcity on contamination levels and provides essential data for evaluating seasonal variations. A total of 29 source water samples (15 from Ekbatan and 14 from Shahid Beheshti DWTPs) and 24 treated water samples (12 from each DWTP) were collected. In addition, 47 groundwater wells within the city were sampled. Sampling sites were selected considering the proximity to hospitals, schools, or other priority areas, water residence time within the network, and the age of infrastructure. A total of 100 samples were collected from different sampling sites in 5-liter polypropylene containers and immediately transported to the laboratory on ice (4 °C) within 2 h of collection and processed for virus concentration and extraction within 4 h; in rare instances where immediate processing was delayed, samples were stored at 4 °C for no longer than 24 h to minimize degradation. Physical and chemical parameters such as residual chlorine (measured using a portable colorimeter, Hach DR/890, following EPA Method 8021), pH (using a calibrated pH meter, Metrohm 827, EPA Method 150.1), and turbidity (using a turbidimeter, Hach 2100Q, EPA Method 180.1) were measured on-site prior to each sample collection. The study was approved by the Ethics Committee of Hamadan University of Medical Sciences (Ref. No: IR.UMSHA.REC.1403.731).

### Virus concentration

Upon thawing samples stored at −20 °C, quality control procedures were conducted to ensure sample integrity prior to nucleic acid extraction. Each sample was inspected for potential physical degradation (*e.g.*, leakage or turbidity changes) and homogenized by gentle inversion. Subsamples were then evaluated spectrophotometrically to verify expected absorbance ratios (A260/A280 ≈ 1.8–2.0) and adequate nucleic acid concentration, confirming sample suitability for downstream PCR analysis. An adsorption-elution method was used to concentrate viruses in water samples as previously described (Junter & Lebrun, 2017). The charge interactions-based method was used to capture viruses from large volumes. Briefly, a 5-liter sample was filtered through a negatively charged membrane (Cat No. 9ZGL-0102Sartobind, Germany). The membrane is coated with a layer of negatively charged cellulose nanofiber facilitating the adsorption of virus particles with a high capture efficiency. This approach is a practical and efficient method for capturing and concentrating viruses in environmental water samples (Junter & Lebrun, 2017). Viruses were eluted using a 70 mL elution buffer consisting of 1.5% beef extract with 0.05 M glycine (BEG) at pH 9.5. Elution buffer was recirculated through the filters for 20 min. The pH was adjusted to 7.0 ± 0.5 with HCl (≥5 mol/L) and centrifuged at 4,000 ×g, at 4 °C for 15 min, the supernatant was discarded, and then the pellet was resuspended in phosphate-buffered saline (PBS). The sample concentrate was stored at −20 °C for subsequent analyses. Chlorine, pH, and turbidity were measured and recorded prior to each sample collection.

### Nucleic acid extraction and cDNA synthesis

Following the manufacturer’s instructions, the viral genome was extracted using an ExgeneTM Viral DNA/RNA kit (Cat No. 302-150 GeneAll, Seoul, Korea). The extracted nucleic acids were quantified using a NanoDrop spectrophotometer (Thermo Fisher Scientific, Waltham, MA, USA), and concentrations typically ranged between 20–40 ng/µL. For each PCR reaction, 2 µL of DNA or cDNA corresponding to approximately 40–80 ng of nucleic acid was used as the template to ensure reproducibility and consistent amplification efficiency. Briefly, 50 µM random hexamer primers and 10 mM dNTPs were added to a 0.2 mL microtube, followed by nuclease-free water to 20 µl and gentle mixing. Then, the mixture was incubated at 65 °C for 5 min and immediately placed on ice briefly, then kept at −20 °C until use. Next, the reverse transcriptase, RNase inhibitor, and remaining reagents are added. After a 5-minute incubation at 25 °C with random hexamer primer, the sample was incubated at 55 °C for 60 min to synthesize cDNA. The resulting cDNA can be directly used in PCR with an appropriate DNA polymerase based on the target length. The primers, target regions, PCR product size, and references used in this study are listed in Table 1.

**Table 1:** Primer sets used for multiplex PCR detection of enteric viruses in water samples. This table presents the primer sequences and assay parameters used to detect various enteric viruses via multiplex and conventional PCR. Viruses are grouped based on assay design, with corresponding forward and reverse primers listed along with their target gene regions, polarity, binding positions, expected product sizes (in base pairs), and literature references. The table includes assays for Astrovirus, Norovirus genogroups I and II, Enterovirus, Hepatitis A and E viruses, Group A and C Rotaviruses, and Adenovirus, forming the molecular basis for the viral surveillance conducted in this study.

**Multiplex PCR group**	**Type of viruses**	**Primers**	**Sequence (5**′** to 3**′)	**Target region**	**Polarity**	**Position**	**Product size (bp)**	**Reference**
1	Astrovirus	PreCAP1	GGACTGCAAAGCAGCTTCGTG	ORF2	+	4,235–4,255	719	Zheng et al. (2016)
		82b	GTGAGCCACCAGCCATCCCT	ORF2	–	4,953-4,934		
	Norovirus GI	G1SKF	CTGCCCGAATTCGTAAATGA	-	+	5,342–5,361	330	Grote (2013)
		G1SKR	CCAACCCAGCCATTATACA	–	–	5671–5653		
	Norovirus GII	COG2F	CAAGAGCCTATGTTCAGGTGGATGAG	–	+	5,003–5,028	387	Grote (2013) and He et al. (2011)
		G2SKR	CCACCTGCATAACCATTGTACAT	–	–	5,389–5,367		
2	Enterovirus	F1	CAAGCACTTCTGTTTCCCCGG	5′-NCR	+	160–180	440	Hong et al. (2010)
		R1	ATTGTCACCATAAGCAGCCA	5′-NCR	–	599–580		
	Hepatitis A virus	P3	TATTTATCTGTCACAGAACAATCAG	capsid	+	2,949–2,973	267	Phan et al. (2005)
		P4	AGGAGGCGGAAGCACTTCATTTGA	capsid	–	3,215–3,190		
3	Group A rotavirus	Beg9	GGCTTTAAAAGAGAGAATTTC	VP7	+	1–28	395	Sharif et al. (2020)
		VP7-1	ACTGATCCTGTTGGCCATCCTTT	VP7	–	373–395		
	Group C rotavirus	G8NS1	ATTATGCTCAGACTATCGCCAC	VP7	+	353–374	352	Thongprachum et al. (2017)
		G8NA2	GTTTCTGTACTAGCTGGTGAAC	VP7	–	683–704		
4	Hepatitis E virus	2s	CCGTCGTCTCAGCCAATGGCGAGC	ORF2	+	6,345–6,368	146	Tamura et al. (2007)
		2as	CTCATGTTGGTTGTCATAATCCTG	ORF2	–	6,490–6,467		
5	Adenovirus	Ad1	TTCCCCATGGCTCACAACAC	Hexon	+	1,834–1,853	482	Rezaei et al. (2012)
		Ad2	CCCTGGTAGCCGATGTTGTA	Hexon	–	2,315–2,296		

### Multiplex PCR

RNA viruses were detected using reverse transcription-polymerase chain reaction (RT-PCR) assays for cDNA formation. After amplification assay was performed, PCR products were visualized through gel electrophoresis. The viral agents for identification were categorized into three multiplex groups and two individual viruses. Group 3 comprised Group B and Group C Rotavirus, group 2 included HAV and Enterovirus, and Group 1 had Astrovirus, Norovirus GI, and Norovirus GII. Adenovirus and HEV were tested separately (Table 1). Each assay included a positive control. Amplification was done in a 25 µL reaction mixture with the PCR Master Mix (Cat no. PB10.13-02, Applied PCR Biosystems). Briefly, the reaction contained 5 µL of a DNA sample or 5 µL of a cDNA, with a final concentration of 1 mM MgCl_2_, 200 mM of each deoxynucleotide triphosphate (dNTP), 1 mM of each primer, and 1 U of Taq DNA polymerase. The PCR conditions included initial denaturation at 95 °C for 2 min, followed by denaturation at 95 °C for 20 s, followed by 25 cycles of annealing at specific temperatures (G1 = 53.8 °C, G2 = 56 °C, G3 = 56 °C, HEV = 60 °C, AdV = 55 °C for 40 s), extension at 72 °C for 50 s, and final extension at 72 °C for 5 min. The PCR products were analyzed by agarose gel electrophoresis using 1% agarose gel (Sinagen, Iran), stained with Safe Stain (Sinagen, Iran), and visualized under UV light using a Gel Doc XR+ system (Bio-Rad, Hercules, CA, USA).

To ensure PCR quality and reliability, several controls were implemented. Positive controls consisted of commercially available synthetic viral RNA/DNA standards (*e.g.*, for AdV, NoV, and EV; sourced from GeneAll, Seoul, Korea) at known concentrations to verify amplification efficiency. Negative controls included no-template controls (NTCs) in each run to detect contamination. The limit of detection (LOD) was established through serial 10-fold dilutions of positive standards, with the lowest detectable concentration being approximately 10^2^ genome copies per reaction for each target, confirmed across three independent runs. All samples were analyzed in duplicate to assess reproducibility, with results considered positive only if both replicates amplified. Primer-dimer formation was checked by examining gel electrophoresis bands for non-specific products below the expected size and, for real-time compatible assays, *via* melt curve analysis to confirm specific amplification peaks.

### Statistical analysis

All statistical analyses were performed using SPSS version 17.0 (IBM, Chicago, IL, USA). As most of the dataset comprised categorical (presence/absence) variables, Chi-square tests were applied to evaluate associations between viral detection rates and sampling sites (*e.g.*, DWTP source, treated water, and groundwater wells). When expected cell frequencies were below five, as observed for low-prevalence viruses such as Rotavirus and Enterovirus, Fisher’s exact test was employed to ensure the validity of comparisons. For continuous physicochemical parameters (*e.g.*, residual chlorine, pH, turbidity), independent samples *t*-tests were used to compare mean values between virus-positive and virus-negative samples. Data are expressed as means ± standard error (SE), and statistical significance was determined using two-tailed tests with a threshold of *p* < 0.05.

## Results

**Figure 1:**
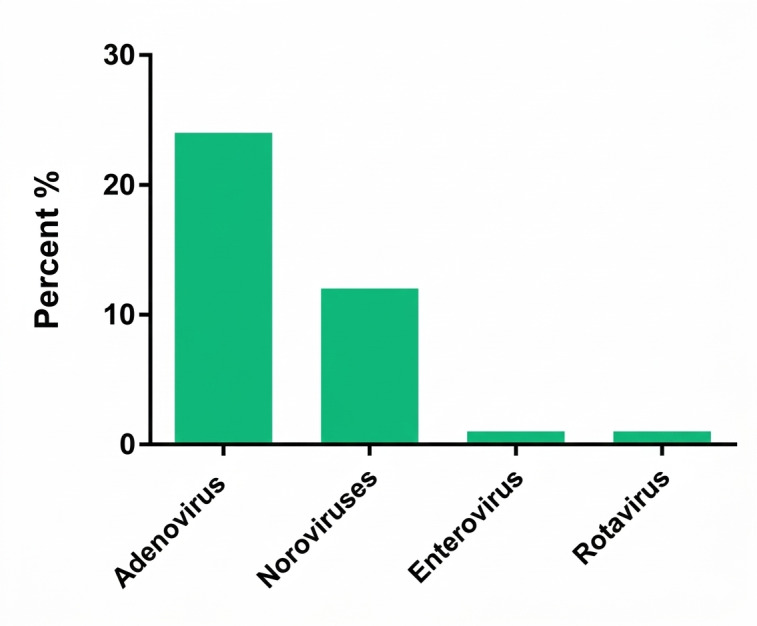
Prevalence of enteric viruses across different water sources in Hamadan, Iran. Each bar represents the number of water samples testing positive for Adenovirus, Norovirus, Rotavirus, and Enterovirus across various sampling sites, including drinking water treatment plant (DWTP) inlets, treated water outlets, and groundwater wells. Viral detection was performed using PCR-based assays, with Adenovirus and Norovirus showing the highest prevalence. The data emphasize greater viral detection in surface water sources (DWTP inlets) compared to treated water and wells, highlighting potential contamination risks in source waters.

**Figure 2:**
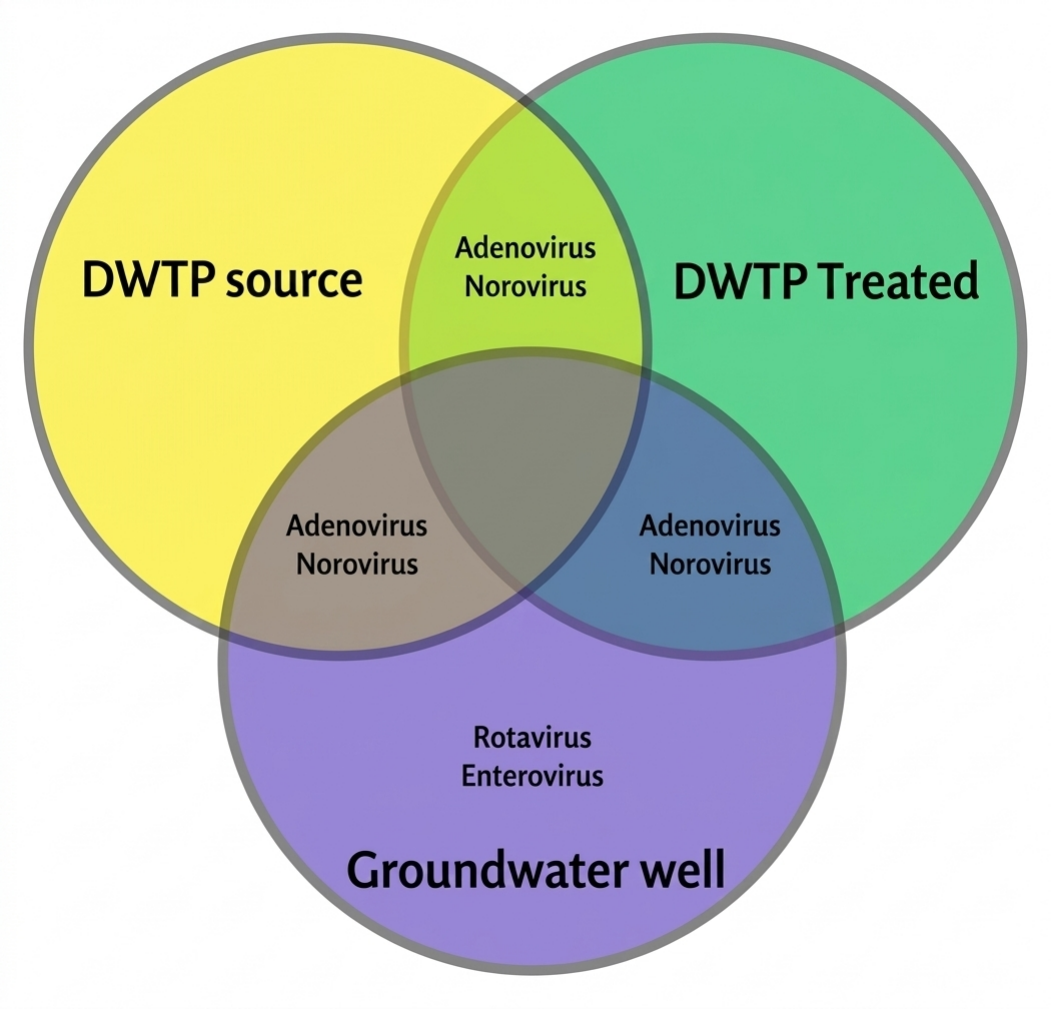
Diversity and distribution of enteric viruses in Hamadan’s Drinking Water System. The variety of enteric viruses detected across different water sampling locations, including Shahid Beheshti and Ekbatan Drinking Water Treatment Plants (inlet and outlet) as well as groundwater wells. Each virus type—Adenovirus, Norovirus, Rotavirus, and Enterovirus—is represented to show its detection frequency per site. The figure emphasizes that Adenovirus was the most widespread across all locations, with surface water sources showing greater viral diversity than treated or groundwater samples. PCR-based multiplex assays were used for virus identification.

This cross-sectional study detected DNA and RNA viruses in the source and treated drinking water in the city of Hamadan. Adenoviruses were detected in 24% (24/100) of samples, with a significant difference in positivity rate between the two DWTPs (*p* < 0.0001 chi-square test), whereas 12% (12/100) of samples tested positive for Norovirus, with a borderline significant difference between locations (*p* = 0.05). Ekbatan DWTP exhibited the highest positivity rates for both Adenoviruses and Noroviruses, with 29.6% (8/27) of samples testing positive for Adenoviruses and 22.2% (6/27) of samples testing positive for Noroviruses. Rotavirus and Enterovirus (EV) were rarely detected, with only 2.1% (1/47) of well samples tested positive for each of the viruses (Fig. 1). No statistically significant association was observed between site and Rotavirus positivity (*p* > 0.05). The groundwater wells had the lowest levels of viral contamination, with 21.3% (10/47) of samples tested positive for Adenoviruses and 6.4% (3/47) for Noroviruses. In contrast, the sources water samples at Ekbatan DWTP had the highest levels of viral contamination, with 46.7% (7/15) of samples tested positive for Adenoviruses and 33.3% (5/15) for Noroviruses. Figure 2 illustrates the comparative prevalence of enteric viruses across the water system.

This cross-sectional study highlights a greater prevalence of AdV and NoV GI at municipal water treatment facilities than in groundwater wells (Table 2). The physical-chemical analyses for chlorine, turbidity, and pH revealed the mean values of 0.77 ± 0.8 mg/L (*n* = 92), 9.13 ± 20.5 NTU (*n* = 92), and 7.56 ± 0.1 (*n* = 100), respectively. The temporal distribution of virus-positive samples across the four sampling months (June–November 2018) is presented in Table 3. Most detections occurred in August and September, accounting for 34 and 14 positive samples, respectively. Only four positive samples were observed in October, and no viruses were detected in November. These findings indicate a temporal decline in viral detection toward late autumn rather than a confirmed seasonal trend, as the study period covered only part of summer and the entire autumn. Regardless of the fact that PCR methods lack infectivity determination, no significant relationship was observed between chlorine residues and the presence or absence of AdV and NoV viruses (*p* > 0.05) (Table 4). These findings suggest potential limitations in disinfection efficacy but do not confirm infectious risks, as viral viability was not assessed. All viral pathogens were only detected in summer, except Adenovirus, which was detected in both summer and autumn.

**Table 2:** Detection frequencies of enteric viruses across source, treated, and groundwater samples. This table summarizes the number of water samples that tested positive and negative for Adenovirus, Norovirus, Rotavirus, and Enterovirus across three sampling categories: source water from DWTP inlets, treated water from DWTP outlets, and groundwater wells. Results are presented separately for Ekbatan and Shahid Beheshti Drinking Water Treatment Plants. The total number of tested samples and virus detections are shown, along with *p*-values calculated using the Chi-square test to assess the statistical significance of virus distribution by sample type. Notably, Adenovirus showed the highest detection rate with a significant association between sampling site and presence. Astrovirus was not detected in any sample.

**Type of virus**		**Sample tested**	**DWTP source**	**DWTP treated**	**Ground water well (n)**	**Total samples**	*p*-value^*^
Adenovirus	Ekbatan-DWTP	Positive	7	1	–	8	<0.0001
		Negative	8	11	–	19	
	Shahid Beheshti- DWTP	Positive	6	0	–	6	
		Negative	8	12	–	20	
	Ground water well	Positive	–	–	10	10	
		Negative	–	–	37	37	
	Total	Positive	13	1	10	24	
		Negative	16	23	37	76	
		Samples	29	24	47	100	
Norovirus	Ekbatan- DWTP	Positive	5	1	–	6	0.05
		Negative	10	11	–	21	
	Shahid Beheshti-WTP	Positive	3	0	0	3	
		Negative	11	12	0	23	
	Ground water well	Positive	–	–	3	3	
		Negative	–	–	44	44	
	Total	Positive	8	1	3	12	
		Negative	21	23	44	88	
		Samples	29	24	47	100	
Rotavirus	Ekbatan- DWTP	Positive	–	–	–	0	>0.05
		Negative	–	–	–	0	
	Shahid Beheshti- DWTP	Positive	–	–	–	0	
		Negative	–	–	–	0	
	Ground water well	Positive	–	–	1	1	
		Negative	–	–	46	46	
	Total	Positive	–	–	1	1	
		Negative	–	–	46	46	
		Samples	–	–	47	47	
Enterovirus	Ekbatan- DWTP	Positive	–	–	–	0	<0.0001
		Negative	–	–	–	0	
	Shahid Beheshti- DWTP	Positive	–	–	–	0	
		Negative	–	–	–	0	
	Ground water well	Positive	–	–	1	1	
		Negative	–	–	46	46	
	Total	Positive	–	–	1	1	
		Negative	–	–	46	46	
		Samples	–	–	47	47	

**Notes.**

* *P*-value was reported based on the Chi-square test.

**Table 3:** Seasonal and monthly detection of enteric viruses in drinking water samples. This table presents the number of water samples testing positive for Adenovirus, Rotavirus, Norovirus, and Enterovirus across five months (June to November) in 2018. Data are also grouped by season-summer (June to September) and autumn (October to November). Adenovirus and Norovirus exhibited significant seasonal patterns, with the majority of positive detections occurring during summer months. Chi-square test *p*-values indicate statistically significant seasonal differences for Adenovirus and Norovirus (*p* < 0.0001), while Rotavirus and Enterovirus showed no significant variation across seasons.

**Type of virus**	**Seasons**		*p*-value^*^	**Type of virus**	**Jun**	**Jul**	**Aug**	**Sep**	**Oct**	**Nov**
	**Summer**	**Autumn**								
Adenovirus	20	4	<0.0001	Adenovirus	20	0	0	0	4	0
Rotavirus	1	0	0.2	Rotavirus	1	0	0	0	0	0
Norovirus	12	0	<0.0001	Norovirus	12	0	0	0	0	0
Enterovirus	1	0	0.2	Enterovirus	1	0	0	0	0	0

**Notes.**

* *P*-value was reported based on the Chi-square test.

**Table 4:** Comparison of residual chlorine levels in adenovirus- and norovirus-positive *versus* negative water samples. This table compares the mean residual chlorine concentrations (mg/L) between water samples that tested positive and negative for Adenovirus and Norovirus. While the mean chlorine level was slightly lower in virus-positive samples, no statistically significant differences were observed for either virus type, as indicated by Chi-square test *p*-values (*p* > 0.05). These results suggest that the presence of residual chlorine alone may not predict viral presence in drinking water samples.

**Type of virus**		**Frequency**	**Mean ± SD (mg/L)**	*p*-value^*^
Adenovirus	Positive	24	0.66 ± 0.5	0.61
	Negative	76	0.77 ± 0.8	
Norovirus	Positive	12	0.74 ± 0.6	0.99
	Negative	88	0.75 ± 0.8	

**Notes.**

* *P*-value was reported based on the Chi-square test.

## Discussion

Increasing viral disease outbreaks necessitate in-depth investigation of the occurrence of waterborne viruses at spatial and temporal spectrum in source waters and the environment (Fong & Lipp, 2005). This study used Reverse Transcription Polymerase Chain Reaction (RT-PCR) to detect viral RNA and PCR for AdV in the water samples. These methods are commonly used for the detection of viruses in aquatic environments (Hamza et al., 2011). It has been successfully used for a national study of viral prevalence in drinking water systems in the United States (Abbaszadegan & Alum, 2016; Abbaszadegan, Lechevallier & Gerba, 2003). RT-PCR offers high sensitivity and specificity, allowing for reliable detection of low viral loads (Watzinger, Ebner & Lion, 2006). However, it can be costly and requires specialized equipment and trained personnel, which may limit its scalability.

Both RNA and DNA viruses were detected in this study. Our findings align with regional patterns in Iran, where enteric viruses exhibit high prevalence and diversity in environmental samples. For example, in Tehran, NoV, RV, AstV, and AdV were detected in 20% of vegetable samples overall (with RV at 23% in phase II farms) and 25% of irrigation water, mirroring our 24% AdV and 12% NoV positivity in Hamadan water sources and suggesting shared contamination pathways *via* sewage-irrigated agriculture (Rafieepoor et al., 2024). In Zahedan, wastewater surveillance revealed near-ubiquitous NoV GII (100%) and high RV (91.66%) and EV (83.33%) rates, with genotypes like NoV GII.P17 and Echovirus 14, supporting our detection of NoV, RV, and EV and indicating year-round circulation potentially exacerbated by inadequate treatment (Nejati et al., 2025). In Hamadan, a 2025 original study reported AdV persistence in 80% of WWTP effluents and 28.6% of DWTP treated water *via* nested PCR, reinforcing the robustness of our multiplex PCR approach for detecting low-level contamination in treated waters and highlighting inefficiencies in conventional treatments like chlorination, as seen in our study (*e.g.*, no significant chlorine-virus correlation) (Owliaee et al., 2025). These comparisons highlight the need for nationwide surveillance, particularly in understudied areas like Hamadan, where seasonal water scarcity may exacerbate viral persistence and transmission risks. This likely reflects the robust environmental stability of AdV, which is resistant to many common disinfectants and can remain infectious in the environment for hours, including on surfaces and medical instruments (Abbaszadegan et al., 2008; CDC, 2024). AdV is regarded as the most resistant pathogen to UV disinfection by monochromatic, low-pressure UV irradiation at 254 nm (Beck et al., 2014). AdV is composed of a DNA genome, a factor that may contribute to its robustness and adaptability. The predominance of AdV aligns with other studies showing its persistence through treatment processes, supporting its use as an index virus indicating risks. AdVs are recognized as significant pathogens associated with various infections, including gastrointestinal disorders, respiratory infections, and eye infections (Jiang, 2006). Myrmel et al. (2006) reported Adenoviruses in 96% of influent and 94% of effluent samples in WWTPs (Myrmel et al., 2006). In their 2023 study, Soltane & Allayeh (2023) reported a 33% removal rate in a WWTP. Among the RNA viruses, NoV was most frequently detected in this study. Kukkula et al. (1999) detected NoV II in drinking water from various sources in Finland. A study in Portugal did not find NoV and HAV RNAs in drinking water; however, HEV was detected (Teixeira et al., 2020). In Spain, Blanco et al. (2017) noticed NoV I and NoV II RNA in high concentrations in drinking water. HAV was identified in treated waters in several municipalities in Colombia, but it wasn’t detected in our study (Peláez et al., 2016).

In this study, Rotavirus detection rates were detected in 1% of sample, which lower compared to those reported by Verheyen et al.. However, differences in detection protocols, sample sizes, and process controls between the studies should be considered. These methodological variations are crucial for interpreting discrepancies in Rotavirus prevalence (Verheyen et al., 2009). Overall, the highest viral detection occurred at the influent of Ekbatan DWTP. These differences may be attributable to variations in treatment trains; for instance, the pre-ozonation step in Shahid Beheshti DWTP could enhance precursor removal and disinfection efficacy compared to Ekbatan’s primary chlorination approach, potentially explaining lower post-treatment detections at Shahid Beheshti.

There was also a strong seasonal trend, with increased viral contamination in the summer months. The summer increase reflects higher recreational activities in source waters, impacting microbial water quality and a challenge to removal efficiency during treatment processes. Overall, viral pathogens in treated drinking water reveal deficiencies in existing water treatment processes to mitigate viral risks. Rachmadi et al. (2020) highlight that chlorine disinfection alone may not be sufficient to achieve the desired level of virus removal in all cases. The presence of viral RNA in the DWTP outlet warrants further investigation, given the low infectious dose of enteric viruses and potential for waterborne transmission (Borchardt et al., 2003) though infectivity and quantitative exposure were not evaluated in this study. This study provides a snapshot of the occurrence of the enteric virus in the city of Hamadan drinking water systems. However, the experimental plan has several limitations in the sampling campaign, such as a limited number of samples from each site, a qualitative nature of data without quantifying viral concentrations, and the detection methodology used without infectivity determination of detected viral particles. These limitations preclude definitive conclusions on public health risks, and future studies should incorporate quantitative methods (*e.g.*, qPCR) and infectivity assays (*e.g.*, cell culture) to better assess implications. The limited sampling period (June–November 2018) represents only a partial seasonal window. Therefore, the apparent summer increase in viral detection should be considered a preliminary observation. The unequal monthly distribution of samples further limits temporal comparisons. Extended, year-round surveillance is needed to confirm these patterns and more accurately characterize seasonal variability. Although the conventional PCR and RT-PCR assays used in this study provided reliable detection of enteric viral genomes, we acknowledge that the application of quantitative real-time RT-PCR would offer enhanced analytical sensitivity and the capacity to estimate viral load. Nevertheless, the primary aim of this investigation was to qualitatively assess the occurrence of enteric viruses across multiple water sources in Hamadan rather than to quantify their concentrations. Moreover, the absence of infectivity assays represents a methodological limitation. The PCR-based approach confirms the presence of viral genomes but cannot determine whether these correspond to viable and infectious viral particles or residual nucleic acids. Consequently, the results presented here should be interpreted as qualitative indicators of viral occurrence rather than direct evidence of infectious risk. Incorporating infectivity testing and quantitative real-time PCR in future studies will provide a more comprehensive understanding of treatment performance and actual public health implications. Detecting viruses in drinking water supplies shows that treatment methodologies must effectively eliminate viral pathogens (World Health Organization, 2016).

## Conclusion

This study provides valuable insights into the occurrence of enteric viruses across drinking water sources serving the city of Hamadan, Iran. Both RNA and DNA human pathogenic viruses were detected, including AdV, NoV, RV, and EV. Viral contamination exhibited distinct seasonal trends, with significantly higher viral occurrence rates in summer. In light of the prevalence of viruses in treated water samples, it is recommended to focus on proper operations and management of the water system for producing high-quality drinking water. This can be accomplished by enforcing proper operation of each step of the process to sustain treatment requirements and to meet regulations. In addition, it is advisable to investigate and identify pollution sources that may impact source water quality. In summary, the outcome of the study suggests potential environmental circulation of enteric viruses, and the waters of Hamadan county’s water supplies should be considered as an indicator of possible contamination by public health agencies, with further quantitative and infectivity studies needed to evaluate risks.

##  Supplemental Information

10.7717/peerj.21173/supp-1 Supplemental Information 1 Raw measurements of enteric virus surveillance in hamadan water sources Raw field and laboratory data collected from various water sources in Hamadan, Iran, as part of an enteric virus surveillance study conducted in 2018. Water samples were obtained from drinking water treatment plant (DWTP) inlets and outlets, as well as groundwater wells, and analyzed using physical-chemical and molecular virology methods.Virus results are reported qualitatively (*e.g.*, “positive”, “0”, or blank), representing detection status per PCR test. These measurements form the basis for summary statistics and figures reported in the manuscript.

## Additional Information and Declarations

### Data Availability

The following information was supplied regarding data availability: The raw data are available in the Supplementary File.
